# Inflammation condition sensitizes Piezo1 mechanosensitive channel in mouse cerebellum astrocyte

**DOI:** 10.3389/fncel.2023.1200946

**Published:** 2023-05-25

**Authors:** Donggyeom Yu, Ajan Ahmed, Jazmine Jayasi, Andres Womac, Olajuwon Sally, Chilman Bae

**Affiliations:** School of Electrical, Computer, and Biomedical Engineering, Southern Illinois University, Carbondale, IL, United States

**Keywords:** mechanosensitive channel, Piezo1 channel, inflammation, astrocyte, mechanotransduction, electrophysiology, wound healing, calcium imaging

## Abstract

Piezo1 mechanosensitive ion channel (MSC) plays a significant role in human physiology. Despite several research on the function and expression of Piezo1 in the nervous system, its electrophysiological properties in neuroinflammatory astrocytes remain unknown. We tested whether astrocytic neuroinflammatory state regulates Piezo1 using electrical recordings, calcium imaging, and wound healing assays on cultured astrocytes. In this study, we determined whether neuroinflammatory condition regulates astrocytic Piezo1 currents in astrocytes. First, we performed electrophysiological recordings on the mouse cerebellum astrocytes (C8-S) under lipopolysaccharide (LPS)-induced neuroinflammatory condition. We found that LPS treatment significantly increased MSC currents in C8-S. The half-maximal pressure of LPS treated MSC currents was left-shifted but the slope sensitivity was not altered by LPS treatment. LPS-induced increase of MSC currents were further augmented by Piezo1 agonist, Yoda1 but were normalized by Piezo1 inhibitor, GsMTx4. Furthermore, silencing Piezo1 in LPS treated C8-S normalized not only MSC currents but also calcium influx and cell migration velocity. Together, our results show that LPS sensitized Piezo1 channel in C8-S astrocytes. These findings will suggest that astrocytic Piezo1 is a determinant of neuroinflammation pathogenesis and may in turn become the foundation of further research into curing several neuronal illnesses and injury related inflammation of neuronal cells.

## Introduction

Mechanotransduction describes the ability of cells to detect, integrate, and convert mechanical stimuli into biochemical signals that directly influence biological functions (Kolahi and Mofrad, 2010). Mechanosensitive ion channels (MSCs) are well characterized biological force sensing systems that initiate a process of mechanotransduction (Martinac, 2012). In eukaryotic cells, MSCs have been associated with various pathological diseases such as cardiovascular physiology (Mingote et al., 2015), muscular dystrophy (Lansman and Franco-Obregon, 2006), neurodegeneration (Song et al., 2019), and cancer (De Felice and Alaimo, 2020).

Piezo1 is a non-selective cation MSC that opens on response to mechanical force and participates in the transmission of biomechanical signals on the cellular membrane in various cell types (Coste et al., 2010; Zhu et al., 2021). Piezo1 can be gated in bleb-attached patches in the absence of the cytoskeleton, suggesting that it is gated according to the “force-from lipids” principle, an evolutionarily conserved gating mechanism (Cox et al., 2017). Aside from mechanical forces, Piezo1 can be chemically activated with Yoda1 (Syeda et al., 2015). Activation of Piezo1 can be inhibited by Grammostola mechanotoxin #4 (GsMTx4), a peptide from spider venom. Furthermore, Piezo1 is essential for cell survival, and mutations in Piezo1 have been linked to cause Xerocytosis (Zarychanski et al., 2012; Bae et al., 2013b; Glogowska et al., 2017), and generalized lymphatic dysplasia (Fotiou et al., 2015; Lukacs et al., 2015).

Neuroinflammation is central to the common pathology of several diseases. However, limited information is known regarding how ion channels are involved during this process. Glial cells express a variety of ion channels from the transient receptor potential vanilloid family, such as Transient receptor potential vanilloid 1 (TRPV1) and Transient receptor potential vanilloid 4 (TRPV4), Ho et al. (2014), voltage-gated potassium channels (Bozic et al., 2018), and sodium channels (Pappalardo et al., 2016). Lipopolysaccharide (LPS) can activate TRP channels such as TRPV1 to regulate astrocyte activation (Yoshida et al., 2016). The TRPV4 channel induces Ca^2+^ influx when activated (Hong et al., 2016) and is abundant in the central nervous system (CNS), including astrocytic membranes (White et al., 2016). Opening of Connexin 43 (Cx43) hemichannel and TRPV4 channels are critical components of mechanosensory Ca^2+^ signaling in rodent brainstem astrocytes, indicating that mechanosensory transduction in astrocytes depend on the interaction between TRPV4 and Cx43 channels in the CNS (Turovsky et al., 2020). K^+^ channels (K_*v*_) were characterized in microglia and K_*v*_ 1.3 was found to be increased by LPS-induced microglia (Kettenmann et al., 1990). Aside from neurons, sodium channels (Na_*v*_) are also expressed in astrocytes and microglia, where their role is still being established (Hossain et al., 2013).

The activation of glial cells, particularly astrocytes, is known to induce neuroinflammation (Park et al., 2018) and measuring the mechanical properties of astrocytes and their MSCs under such conditions is crucial. Although Piezo1 has been implicated in inflammatory responses (Velasco-Estevez et al., 2020b; Shinge et al., 2022) the precise mechanisms remain unclear. Moreover, despite several studies investigating the function and expression of Piezo1 in the nervous system (Song et al., 2019; Qu et al., 2020; Velasco-Estevez et al., 2020a), the electrophysiological properties of Piezo1 in neuroinflammatory astrocytes are not well-established. To address this research gap, we conducted electrophysiological recordings, calcium imaging, and wound healing assays on cultured Mouse cerebellum astrocytes (C8-S) astrocytes to determine if neuroinflammatory conditions regulate Piezo1 currents. Our study aimed to establish the electrophysiological properties of Piezo1 in neuroinflammatory astrocytes and provide insights into the potential role of Piezo1 in neuroinflammation.

## Materials and methods

### Cell culturing

C8-S were obtained from ATCC (CRL-2535, Manassas, VA, USA) and cultured according to the manufacturer’s instructions. Briefly, cells were cultured in Dulbecco’s modified Eagle’s medium with 4.5 g/L glucose and L-glutamine (Lonza, Walkersville, MD, USA), and 10% fetal calf serum and 1% penicillin-streptomycin (Gibco, Billings, MT, USA) on poly-L-lysine coated cover slips or culture flasks at 37°C under 5% CO_2_. To simulate a neuroinflammation model (Sheng et al., 2003; Qin et al., 2007; Kim et al., 2015), cells were treated with and without 100 ng/ml LPS (Carlsbad, CA, USA) 24 h after seeding and allowed to incubate for 24 h.

### Electrophysiology

Mechanosensitive ion channel currents were recorded using cell-attached and outside-out patches under voltage clamp mode, as previously described in our studies (Zarychanski et al., 2012; Bae et al., 2013a,b; Glogowska et al., 2017). Briefly, channel currents were recorded using a Multiclamp 700B amplifier, DigiDATA, and pClamp software (version 10.6) (Molecular Device, San Jose, CA, USA) with a sampling rate of 10 kHz and a filtering rate of 2 kHz and the bath solution [in mM: 145 NaCl, 5 KCl, 1 MgCl_2_, 2.5 CaCl_2_, and 10 Hepes (pH 7.4, adjusted with NaOH)]. The recording pipettes (1–2 MΩ) were filled with internal solution [in mM: 150 KCl, 0.5 MgCl_2_, 0.25 EGTA, 10 Hepes (pH 7.3, adjusted with KOH)]. Mechanical stimulation was applied through the recording pipette by suction for cell-attached configuration or positive pressure for outside-out configuration using a high-speed pressure clamp system (HSPC-2, ALA scientific instruments, Farmingdale, NY, USA). During the current recording, 10 μM Yoda1 (CAS No.: 448947-81-7, Millipore Sigma, Burlington, MA, USA) or 10 μM GsMTx4 (donated from Dr. Frederick Sachs’ Lab in SUNY Buffalo) was perfused. In the cell-attached configuration, the current vs. voltage (I-V) relationship was determined by measuring the current response to −40 mmHg pressure at holding voltages ranging from −60 mV to 60 mV (30 mV step). The current vs. pressure (I-P) relationship was determined by measuring the current response to suction pressures between −10 mmHg and −80 mmHg (10 mmHg step). In the outside-out configuration, the I-V relationship was measured at holding voltages ranging from −60 mV to 60 mV (30 mV step) using + 30 mmHg pressure. The I-P relationships were determined at a holding voltage of + 60 mV using pressures ranging from 10 mmHg to 70 mmHg (10 mmHg step).

### Piezo1 siRNA transfection

Piezo1 siRNA (Dharmacon Inc., Lafayette, CO, USA) was used to down-regulate mRNA. Mouse Piezo1-targeting siRNA sequences were 5′-GAAAGAGAUGUCACCGCUA-3′, 5′-GCAUCAACUUCCAUCGCCA-3′, and 5′-AAAGACAGAUGAA GCGCAU-3′. Piezo1 siRNA was treated for 24–48 h, followed by LPS (100 ng/ml) for 24 h. And then, 10 μl of mouse Piezo1 siRNA were mixed with 190 μl of Opti-MEM reduced serum medium (Thermo Fisher Scientific, U.S). Simultaneously, 4 μl of DharmaFECT Transfection Reagent (Thermo Fisher Scientific, U.S) was mixed with 196 μl of Opti-MEM reduced serum medium in a separate centrifuge tube. After dilution with antibiotic free medium, the media was replaced and incubated with the siRNA mixture for 48 h.

### Ca^2+^ imaging

To perform calcium imaging, cells were treated with 3 μM Cal-520AM dye (Abcam, MA) for 90 min at 37°C followed by 30 min at 22°C in Hank’s balanced salt solution supplemented with 10 mM glucose and 25 mM HEPES. The imaging was carried out using a confocal laser scanning microscope (FV3000; Olympus, Tokyo, Japan) in accordance with the manufacturer’s instructions. Four experimental groups were used: Control, LPS, siRNA, and LPS + siRNA. After recording the baseline fluorescent intensity (F_*o*_) for 30 s, the bath solution was treated with 10 μM Yoda1 or DMSO (control) while recording the change in fluorescence for all experimental groups. Fluorescence intensity was analyzed using ImageJ, and the difference between the peak and baseline intensities (ΔF) was normalized by the baseline and plotted using GraphPad Prism.

### Wound healing assay

For the cell migration assay, approximately 4,000 cells/mm^2^ were seeded in a 35 mm petri dish coated with poly-D-lysine. A wound was created either with a micropipette tip or an Ibidi micro-insert (Ibidi, Gräfelfing, Germany) 6–8 h after seeding. The cells were then treated with or without 20 nM LPS. The entire wound region was analyzed using ImageJ. The cell migration velocity was determined by dividing the difference between the initial and final wound widths by the elapsed time of 12 and 24 h, respectively, using the wound area and width values.

### Statistical analysis

The data are presented as mean ± standard error of the mean (SEM), where n represents the number of cells and N represents the number of experiments. The data were analyzed using GraphPad Prism (version 9) and Origin (version 2020b) software. The Mann–Whitney *t*-test was used to compare the means of current amplitude, fluorescent intensity, and velocity between two groups, while Ordinary One-Way ANOVA was used for comparing multiple groups. Statistical significance was considered at *p* < 0.05 (*), or *p* < 0.01 (^**^).

## Results

### LPS increased MSC currents in C8-S astrocytes

**FIGURE 1:**
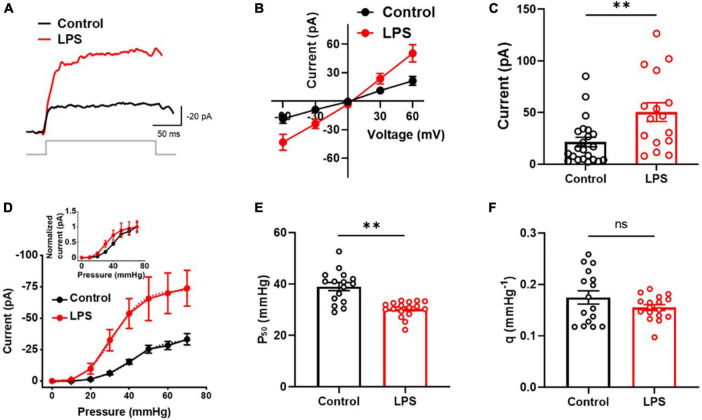
Mechanosensitive channel (MSC) currents were sensitized by LPS in C8-S astrocytes in outside-out configuration. **(A)** Representative traces of MSC current at 30 mmHg and –60 mV. **(B)** Current vs. voltage response at 30 mmHg. **(C)** Summary of experiments for panel **(B)** at 30 mmHg at 60 mV. Control: *n* = 22; LPS: *n* = 16. **(D)** Current vs. pressure response at –60 mV. LPS increased MSC currents significantly and left-shifted the I-P curve (inset). Control: *n* = 16; LPS *n* = 18. **(E)** Half-maximal pressure (P_50_) of panel **(D)**. LPS sensitizes MSC. Control: *n* = 16; LPS: *n* = 17. **(F)** Slope sensitivity of panel **(D)**. Control: *n* = 16, black; LPS: *n* = 17, red; ^**^p < 0.01 and ns: no significance analyzed by unpaired Mann–Whitney *t*-test.

In this study, we investigated the effect of inflammatory conditions on Piezo1 current in astrocytes. Patch clamp recordings were performed on C8-S astrocytes that were exposed to LPS for 24 h. Our findings revealed that LPS treatment increased MSC currents in C8-S astrocytes at −60 mV holding voltage and 30 mmHg pressure (Figure 1A). The currents vs. voltage relationship during outside-out configuration was linear between −60 and + 60 mV with a positive holding pressure of 30 mmHg and a reversal potential of approximately 0 (Figure 1B). Moreover, LPS-induced MSC currents were significantly higher compared to controls at 60 mV with 30 mmHg pressure (*p* = 0.0042; Control: 21.5 ± 4.6 pA; LPS: 50.4 ± 9.1 pA) (Figures 1B, C). Further analysis of the currents vs. pressure curve showed that LPS treatment left-shifted the curve (Figure 1D). The P_50_ was significantly reduced by LPS treatment, indicating sensitization of the MSC channels to mechanical stimuli (*p* < 0.01; Control: 39.0 ± 1.6 mmHg; LPS: 30.3 ± 0.8 mmHg) (Figure 1E). However, the slope sensitivity remained unaltered, indicating no changes in the opening size of the channels (*p* = 0.53; Control: 0.17 ± 0.01 mmHg^–1^; LPS: 0.16 ± 0.01 mmHg^–1^) (Figure 1F). These results indicate that LPS treatment sensitizes MSC channels in C8-S astrocytes to mechanical stimuli without affecting their conductance properties.

### LPS-induced increase of MSC currents in C8-S astrocytes were inhibited by GsMTx4 and silencing Piezo1 but enhanced by Yoda1

**FIGURE 2:**
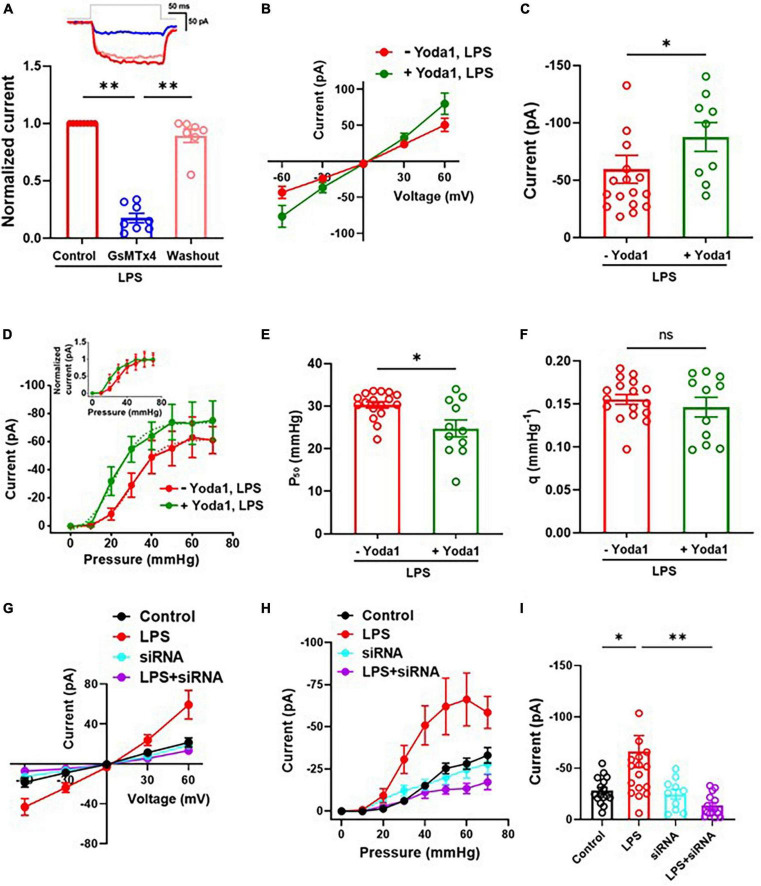
LPS-induced increase of MSC currents were Piezo1 dependent. **(A)** Inhibitory effects of GsMTx4 on MSC currents in LPS-treated C8-S in the outside-out configuration. Control (*n* = 8, red), GsMTx4 (*n* = 8, blue), and washout (*n* = 7, pink). The inset depicts a representative trace. **(B–F)** Effects of Yoda1 on MSC currents. **(B)** Current vs. voltage response at 30 mmHg. –Yoda1, LPS: *n* = 16 and + Yoda1, LPS: *n* = 9. **(C)** Summary of panel **(B)** at –60 mV. *p* = 0.03. **(D)** Current vs. pressure (I-P) response at –60 mV in LPS-treated C8-S. –Yoda1, LPS: red, *n* = 17 and + Yoda1, LPS: green, *n* = 11. **(E)** Half-maximal pressure (P_50_) of panel **(D)**. *p* = 0.03; –Yoda1, LPS: red, *n* = 17 and + Yoda1, LPS: green, *n* = 11. **(F)** Slope sensitivity of panel **(D)** (*p* = 0.82). **(G–I)** Effects of silencing Piezo1 on MSC currents in LPS-treated C8-S. **(G)** Current vs. voltage response at 30 mmHg. **(H)** I-P response at –60 mV in LPS-treated C8-S. **(I)** Summary of panel **(H)**. Peak MSC currents at –60 mV with 50 mmHg in outside out configuration. Control: black, *n* = 16; LPS: red, *n* = 16; siRNA: cyan, *n* = 10; LPS + siRNA: purple, *n* = 14; *p* = 0.03 between Control and LPS and *p* < 0.01 between LPS and LPS + siRNA by one-way ANOVA test. *p < 0.05, ^**^p < 0.01, and ns: no significance.

To investigate whether the LPS-induced increase of MSC currents in C8-S astrocytes was mediated by PIEZO1 currents, we conducted outside-out patch clamp recordings with the perfusion of a Piezo1 antagonist (GsMTx4) and agonist (Yoda1) on LPS-treated C8-S astrocytes. The perfusion of 10 μM GsMTx4 reduced MSC currents at −60 mV holding voltage with 30 mmHg in LPS-treated C8-S cells, and this inhibition was reversible following peptide washout (Control: 1; GsMTx4: 0.18 ± 0.04; Washout: 0.89 ± 0.06) (Figure 2A). Furthermore, 10 μM Yoda1 increased the LPS-induced increase of MSC currents in C8-S cells at −60 mV holding voltage (*p* = 0.0336; −Yoda1, LPS: −59.6 ± 12.2 pA; + Yoda1, LPS: −87.7 ± 12.6 pA) (Figures 2B, C). The LPS-induced increase of MSC currents vs. pressure curve was left-shifted by Yoda1 compared to LPS alone, indicating sensitization of the MSC channels (−Yoda1, LPS: red and + Yoda1, LPS: green) (Figure 2D and its inset). The P_50_ was significantly reduced in the LPS + Yoda1 group, but the slope sensitivity remained unchanged (*p* = 0.03; Control: 30.3 ± 0.8 mmHg; LPS + Yoda1: 24.8 ± 2.0 mmHg) (Figure 2E) (*p* = 0.82; Control: 0.16 ± 0.01 mmHg^–1^; LPS + Yoda1: 0.15 ± 0.01 mmHg^–1^) (Figure 2F). To confirm whether the LPS-induced increase of MSC currents was due to specific Piezo1 currents, we conducted patch clamp recordings on siRNA-treated and LPS + siRNA-treated C8-S cells (Figures 2G–I). Our results showed that the LPS-induced increase of MSC currents was significantly reduced by Piezo1 siRNA treatment in LPS-treated C8-S cells, while siRNA itself did not alter the MSC current compared to its control (Control: −28.2 ± 3.2 pA, black; LPS: −66.3 ± 15.6 pA, red; siRNA: −24.6 ± 4.8 pA, cyan; LPS + siRNA: −13.5 ± 2.9 pA, purple; *p* = 0.03 between Control and LPS and *p* < 0.01 between LPS and LPS + siRNA) (Figure 2I).

### Calcium influx was increased in LPS-treated C8-S but normalized by silencing Piezo1

**FIGURE 3:**
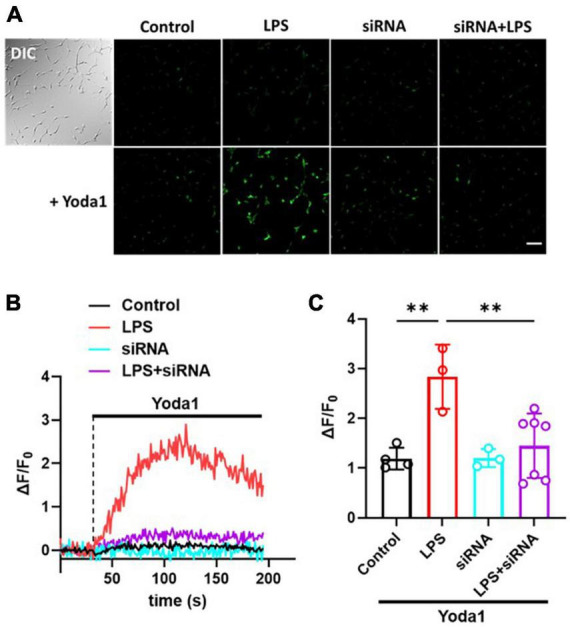
LPS-induced increase of Ca^2+^ influx was inhibited by silencing Piezo1 in C8-S. **(A)** The calcium imaging following 10 μM Yoda1 treatment on C8-S. Scale bar = 100 μm. **(B)** Relative Ca^2+^ sensitive fluorescence time course in C8-S cells pretreated with LPS, siRNA, or LPS + siRNA. **(C)** Summary of relative fluorescence changes from C8-S. Every dot represents the average of all the cells in each individual experiment. Control: *N* = 4, *n* = 177, black; LPS: *N* = 3, *n* = 131, red; siRNA: *N* = 3, *n* = 132, cyan; LPS + siRNA: *N* = 7, *n* = 235, purple; *p* < 0.01 between Control and LPS and *p* < 0.01 between LPS and LPS + siRNA by one-way ANOVA test. ^**^p < 0.01.

To confirm whether the LPS-induced increase of MSC currents in C8-S astrocytes was mediated by Piezo1 channels, we conducted calcium imaging experiments. C8-S cells were treated with LPS for 24 h and then either treated with or without siRNA. Next, 10 μM Yoda1 was perfused on four different groups of C8-S cells [Control (i.e., DMSO), siRNA, LPS, and LPS + siRNA] during calcium imaging (Figure 3). Figure 3A shows representative images of each experimental group after 10 μM Yoda1 treatments. There were no observable differences in fluorescent intensity between groups before Yoda1 treatment and after adding DMSO (Figure 3A; Supplementary Figure 1). In the siRNA group, no calcium influx was observed during Yoda1 treatment. In LPS-treated C8-S cells, perfusion of 10 μM Yoda1 significantly increased calcium influx. However, this increase was normalized in the LPS + siRNA group (Control: 7.5 ± 0.7, black; LPS: 19.5 ± 2.6, red; siRNA: 7.3 ± 0.6, cyan; LPS + siRNA: 8.8 ± 1.5, purple; *p* < 0.01 between Control and LPS and *p* < 0.01 between LPS and LPS + siRNA) (Figures 3B, C). These results confirm that the LPS-induced increase in calcium influx is mediated by Piezo1 channels in C8-S astrocytes.

### Piezo1 regulates cell migration in LPS-treated C8-S

**FIGURE 4:**
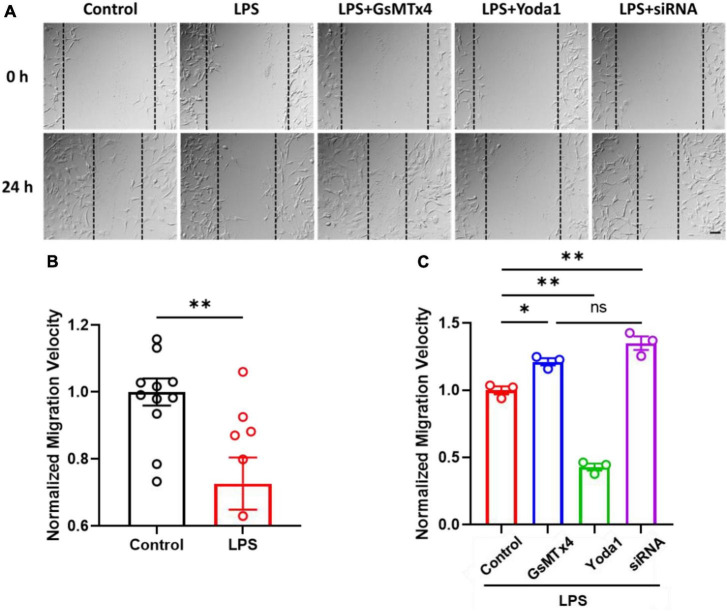
LPS-treated C8-S migration velocities were regulated by Piezo1 channel. **(A)** Representative images of wound healing assays, taken at 0 h and 24 h. Scale bar = 50 μm. Cell density: 4,000 cells/mm^2^. **(B)** Normalized migration velocity of C8-S with or without treatment of LPS. Control: *N* = 11, black; LPS: *N* = 6, red; *p* = 0.04 by unpaired Mann–Whitney *t*-test. **(C)** Normalized migration velocity on LPS-treated C8-S with co-treatment of Piezo1 modulators (10 μM GsMTx4, 10 μM Yoda1, or siRNA). LPS (Control): red, *N* = 3; LPS + GsMTx4: blue, *N* = 3; LPS + Yoda1: green *N* = 3; LPS + siRNA: purple, *N* = 3. *p* = 0.01 between LPS and LPS + GsMTx4; *p* < 0.01 between LPS and LPS + siRNA; and *p* < 0.01 between LPS and LPS + Yoda1. Data were analyzed by ordinary one-way ANOVA test. There was no significant difference between LPS + GsMTx4 and LPS + siRNA. *p < 0.05, ^**^p < 0.01, and ns: no significance.

Finally, in order to investigate the effect of LPS on C8-S migration, we conducted a wound healing assay. Our results revealed that LPS-treated C8-S exhibited a significant reduction in migration velocity at 24 h after wounding compared to control cells (Control: 1.00 ± 0.04; LPS: 0.72 ± 0.08; *p* = 0.003) (Figures 4A, B). Moreover, Yoda1 treatment on LPS-treated C8-S further decreased the migration velocity but inhibition or silencing of the Piezo1 channels in LPS-treated C8-S significantly increased the migration velocity compared to LPS-only treated cells [LPS (Control): red; LPS + GsMTx4: blue; LPS + Yoda1: green; LPS + siRNA: purple. *p* = 0.01 between LPS and LPS + GsMTx4; *p* < 0.01 between LPS and LPS + siRNA; and *p* < 0.01 between LPS and LPS + Yoda1] (Figure 4C). Taken together, these findings suggest that LPS reduces C8-S migration velocity in a Piezo1-dependent manner.

## Discussion

Astrocytes are the most abundant cell type in the CNS. Although astrocytes are not considered to be electrically excitable like neurons (Pappalardo et al., 2016), studies have reported that astrocytes participate in mechanosensory signaling by responding to external stimuli (Turovsky et al., 2020). In this study, we determined whether neuroinflammatory conditions modify biophysical and electrophysiological properties of Piezo1 in astrocytes. To do that, we performed patch-clamp electrophysiology, calcium imaging, wound healing assay to determine how Piezo1 in C8-S astrocytes are affected when stimulated with LPS.

At present, there is no known association between the C8-S cell line and Piezo1. Although it is still possible that Piezo1 activity may vary in primary astrocytes, a previous study (Velasco-Estevez et al., 2020b) reported results similar to ours, indicating that LPS increases Piezo1 expression in primary mouse cortical astrocytes. This discovery sparked our interest in investigating whether cultured astrocytes are also impacted by LPS, particularly with regard to their electrophysiological and biophysical characteristics. Therefore, our study has the potential to establish a model of cultured astrocytes for researching mechanosensitive channels.

Several studies have examined the role of Piezo1 channels in inflammation, with LPS treatment increasing Piezo1 expression in human endothelial cells and Piezo1 knockdown reducing the inflammatory response to LPS (Liu et al., 2022). In a mouse model of acute lung injury, inhibition of Piezo1 reduced inflammation and improved lung function (Zhang et al., 2021), while Piezo1 knockdown decreased the release of proinflammatory cytokines in mouse macrophages (Leng et al., 2022). The effect of inflammation on Piezo1 expression has been investigated, with some studies indicating direct regulation and others suggesting modulation of Piezo1 channels (Chung et al., 2016; Velasco-Estevez et al., 2020b; Lee et al., 2021; Dayawansa et al., 2022). Our study on LPS-treated astrocytes found that inflammation increased Piezo1 sensitivity, as evidenced by increased Piezo1 current and calcium influx regulated by Piezo1 modulators Yoda1, GsMTx4, and silencing Piezo1, compared to non-treated cells. These results align with a previous study on mouse cortical astrocytes (Velasco-Estevez et al., 2020b) which demonstrated LPS-induced upregulation of Piezo1 expression.

Piezo channels can be activated by membrane tension or membrane tethering filaments. In our study, inflammation sensitized Piezo1 channels, evidenced by a leftward shift in the current-pressure curve, without changing the channel’s opening dimensions. In addition to upregulating Piezo1, inflammation may alter the microenvironment of Piezo1 channels, including membrane stiffness and residual tension or linking filaments. More research is required to understand the underlying mechanism by which inflammation sensitizes Piezo1 in astrocytes and the relationship between inflammation, mechanosensitive channel expression, and cellular responses to mechanical stimuli, as other channels may be impacted, as evidenced by Yoda1 and GsMTx4’s influence on control cell migration (Supplementary Figure 2).

Piezo1 channels play important roles in regulating astrocyte functions (Velasco-Estevez et al., 2020b; Chi et al., 2022). Piezo1 channels are essential for astrocyte activation and synaptic transmission in hippocampal neurons (McNeill et al., 2021). Astrocytic Ca^2+^ activities were enhanced in a mouse model of Alzheimer’s disease, which was associated with increased expression of the Piezo1 channel (Gerasimov et al., 2021). In another study, Piezo1 expression was upregulated in astrocytes in response to LPS-induced inflammation *in vitro* (Leng et al., 2022). However, electrophysiological properties of astrocytic Piezo1 under inflammation conditions is barely understood. Our studies confirmed that piezo1 current was increased significantly in LPS-treated astrocytes compared to non-inflammatory conditions. These results support the role of Piezo1 in mediating inflammatory diseases and suggest that further research is needed to fully elucidate the underlying mechanisms.

Calcium signaling process in astrocytes regulates inflammation (Ricci et al., 2009; Liu et al., 2020). Several studies have examined calcium signaling in astrocytes under inflammation conditions using calcium imaging techniques. The administration of LPS *in vivo* and *in vitro* led to increased intracellular calcium levels and induced calcium oscillations in astrocytes, resulting in increased astrocytic calcium signaling and synchronization as well as the release of pro-inflammatory cytokines and other inflammatory mediators (Velasco-Estevez et al., 2020b; Bakaeva et al., 2021; Bobermin et al., 2022). LPS treatment increased intracellular calcium levels and induced calcium oscillations in astrocytes *in vitro*, which was associated with the release of pro-inflammatory cytokines (Batista et al., 2019). In our study, we confirmed that calcium influx through Piezo1 was enhanced in LPS-treated astrocytes.

Piezo1 channels influence the migration of diverse cell types (Yu et al., 2021). However, past research shows contradictory results on the effect of Piezo1 on cell migration. Some studies suggest that silencing Piezo1 leads to an increase in cell migration. In non-small cell lung cancer cells, silencing of either Piezo1 or Piezo12 genes resulted in a notable increase in cell migration *in vitro* and tumor growth *in vivo* (Huang et al., 2019). On the contrary, in glioblastoma, it has been reported that Piezo1 expression is upregulated in highly migratory tumor cells and that Piezo1 knockdown inhibits tumor cell migration (Yu et al., 2021). In endothelial cells, Piezo1-mediated Ca^2+^ influx is involved in the regulation of cell migration, and inhibition of Piezo1 reduces cell migration (Liu et al., 2020). The conflicting results of these studies suggest that Piezo1-dependent cell migration is significantly influenced by the cell type and experimental conditions. Our study investigated the influence of Piezo1 on the migration velocity of LPS-treated C8-S astrocytes and revealed a significant reduction in astrocyte migration in the presence of LPS.

The main objective of this research is to investigate the relationship between Piezo1 and inflammation in astrocytes. Our study found that the Piezo1 current and Piezo1-dependent calcium influx and cell migration were significantly increased in LPS-treated astrocytes when compared to non-inflammatory conditions. Overall, our findings highlight the crucial role of Piezo1 as a mediator of neuroinflammation and suggest that targeting this ion channel could serve as a viable strategy for preventing or treating neuroinflammation.

## Data availability statement

The raw data supporting the conclusions of this article will be made available by the authors, without undue reservation.

## Author contributions

DY conducted most of the experiments and contributed to the experimental design, manuscript editing, and data analysis. AA contributed to the experimental design, manuscript editing, and data analysis. JJ, AW, and OS conducted part of the experiments in Figures 3, 4. CB designed all experiments, wrote the manuscript, and supervised all aspects of the work. All authors contributed to the article and approved the submitted version.

## Supplementary material

Supplementary Figure 1 Time-lapse calcium imaging on C8-S cells before and after administering 10 μM Yoda1, with images displayed every 25 s. Scale bar indicates 100 μm.

Supplementary Figure 2 Wound healing assay on C8-S cells. **(A)** Representative images of wound healing assays, taken at 0 h and 24 h. Scale bar = 50 μm. **(B)** Migration velocity of C8-S with treatment of 10 μM GsMTx4 or 10 μM Yoda1. Control: black, *N* = 6; GsMTx4: blue, *N* = 3; Yoda1: green, *N* = 3; *p* < 0.01 between Control and GsMTx4 and *p* = 0.01 between Control and Yoda1. Data were analyzed by ordinary one-way ANOVA test.
