# Supplementary figures and images for: Inflammation condition sensitizes Piezo1 mechanosensitive channel in mouse cerebellum astrocyte
Source: Front Cell Neurosci. 2023 May 25;17:1200946. doi: 10.3389/fncel.2023.1200946 (PMC10248153; doi:10.3389/fncel.2023.1200946)

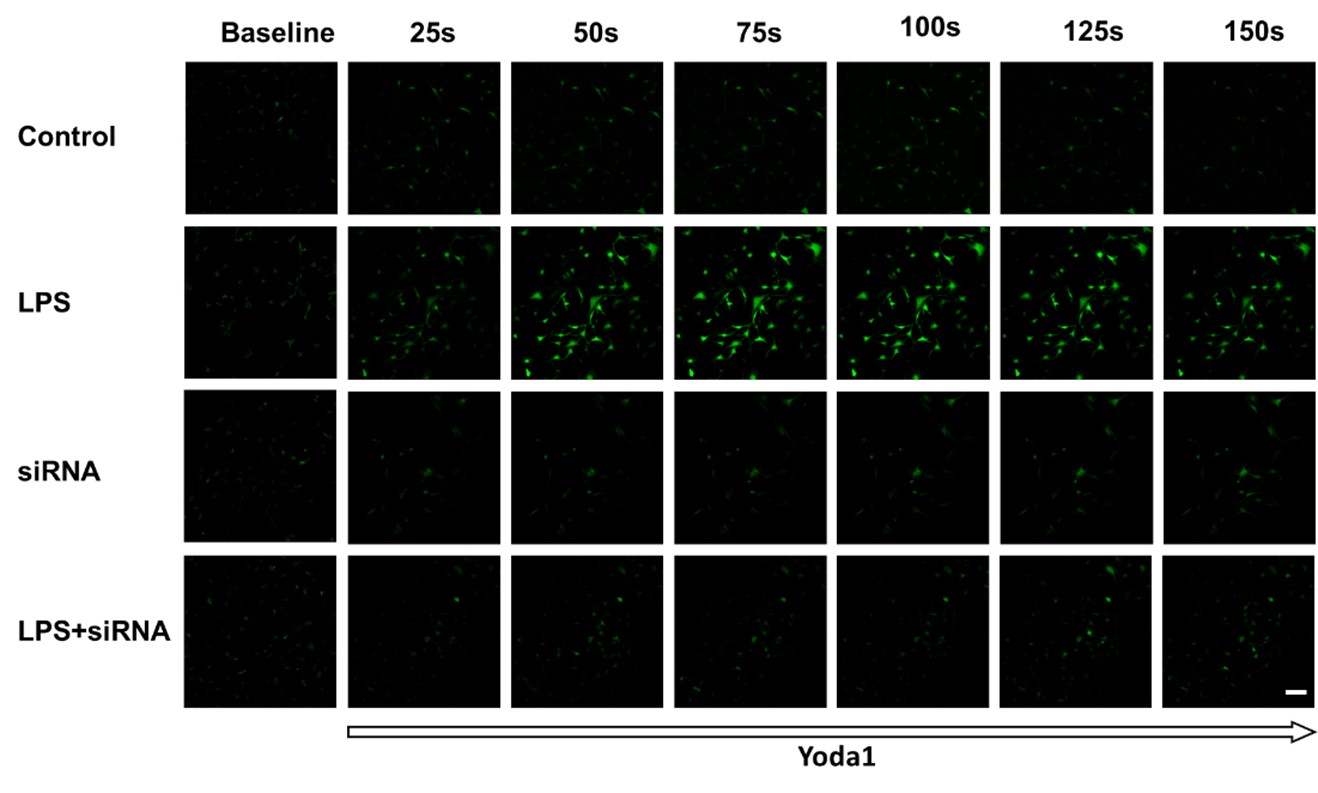

Supplement: Supplementary Figure 1 — Time-lapse calcium imaging on C8-S cells before and after administering 10 μM Yoda1, with images displayed every 25 s. Scale bar indicates 100 μm. [file Image_1.JPEG]

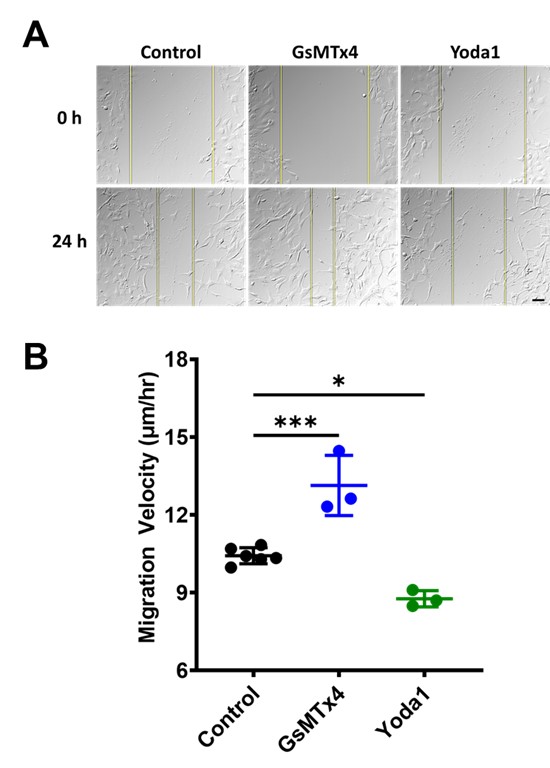

Supplement: Supplementary Figure 2 — Wound healing assay on C8-S cells. (A) Representative images of wound healing assays, taken at 0 h and 24 h. Scale bar = 50 μm. (B) Migration velocity of C8-S with treatment of 10 μM GsMTx4 or 10 μM Yoda1. Control: black, N = 6; GsMTx4: blue, N = 3; Yoda1: green, N = 3; p < 0.01 between Control and GsMTx4 and p = 0.01 between Control and Yoda1. Data were analyzed by ordinary one-way ANOVA test. [file Image_2.JPEG]
